# Risk Assessment Method for CPS-Based Distributed Generation Cluster Control in Active Distribution Networks Under Cyber Attacks

**DOI:** 10.3390/s25196053

**Published:** 2025-10-01

**Authors:** Jinxin Ouyang, Fan Mo, Fei Huang, Yujie Chen

**Affiliations:** 1 Sate Key Laboratory of Power Transmission Equipment Technology, Chongqing University, Chongqing 400044, China; 202311131171t@stu.cqu.edu.cn; 2 State Grid Chongqing Power Company Power Science Research Institute, Chongqing 400044, China; koukoujiangqwq1@163.com; 3 State Grid Xiamen Electric Power Supply Company, Xiamen 361004, China; xm_chenyujie@fj.sgcc.com.cn

**Keywords:** cyber–physical system, active distribution network, DG cluster control, cyber attack, security assessment

## Abstract

In modern power systems, distributed generation (DG) clusters such as wind and solar resources are increasingly being integrated into active distribution networks through DG cluster control, which enhances the economic efficiency and adaptability of the DGs. However, cyber attacks on cyber–physical systems (CPS) may disable control links within the DG cluster, leading to the loss of control over slave DGs and resulting in power deficits, thereby threatening system stability. Existing CPS security assessment methods have limited capacity to capture cross-domain propagation effects caused by cyber attacks and lack a comprehensive evaluation framework from the attacker’s perspective. This paper establishes a CPS system model and control–communication framework and then analyzes the cyber–physical interaction characteristics under DG cluster control. A logical model of cyber attack strategies targeting DG cluster inverters is proposed. Based on the control topology and master–slave logic, a probabilistic failure model for DG cluster control is developed. By considering power deficits at cluster point of common coupling (PCC) and results in internal network of the DG cluster, a physical consequence quantification method is introduced. Finally, a cyber risk assessment method is proposed for DG cluster control under cyber attacks. Simulation results validate the effectiveness of the proposed method.

## 1. Introduction

The increasing share of distributed generation (DG), represented by wind and solar power, is accelerating the evolution of active distribution networks toward DG-centered architectures. Two primary control strategies are adopted for DG management in such networks: centralized and decentralized DG control [1]. Centralized DG control follows a one-to-many configuration, but is prone to interference and suffers from low reliability. In contrast, decentralized DG control adopts a one-to-one structure, allowing individual regulation via dedicated controllers, yet lacks communication between controllers, which prevents effective coordination and optimization among DGs. Consequently, the third one, DG cluster control, has been widely applied [2,3]. In this scheme, a supervisory center coordinates multiple DG clusters, enabling regionalized management of nearby resources while reducing the need for extensive communication infrastructure and supporting plug-and-play integration. Compared to centralized control, DG cluster control offers superior reliability and cost-effectiveness, and is therefore chosen as the focus of this study.

Establishing a cyber–physical system (CPS) for active distribution networks can support the implementation of DG cluster control, enhancing its effectiveness and reliability [4,5,6]. In the power sector, CPS integrates modern communication technologies with advanced control strategies by building a communication network between the physical grid and the dispatch center. This allows the dispatch center to monitor the physical system in real time and issue control commands efficiently [7,8]. A conventional distribution CPS typically adopts a hierarchical architecture, consisting of the master control center, substations, terminal units, and physical equipment [9,10,11]. CPS data are transmitted through carriers such as optical fiber and Ethernet, and the transmission process must comply with international cybersecurity standards [12,13].

In active distribution networks, the CPS extends the conventional framework by enabling the master control center to directly manage DG units. However, the strong coupling between the cyber and physical layers also introduces cyber attack surfaces that hackers can exploit to disrupt normal grid operation between the master control center and DG units [14,15]. Although diverse detection methods against cyber attacks have been proposed in the literature, such as data-driven approaches [16], graph-theoretical methods [17], and time-series forecasting techniques [18], CPS in active distribution networks still face significant risks of cyber intrusions. In particular, cyber attacks targeting the DG cluster may disable the dispatch center’s ability to coordinate DGs, posing serious threats to the secure and stable operation of the active distribution networks [19,20].

Most existing studies on CPS security assessment in active distribution networks rely on mathematical modeling to analyze the physical consequences of cyber attacks and evaluate the network’s disturbance resilience [21,22,23]. Regarding the modeling of attack propagation, ref. [24] developed a CPS reliability evaluation model based on generalized Petri nets to improve computational efficiency, while ref. [25,26,27] proposed component-based models that consider system coupling and topology. Ref. [28] analyzed failure patterns of system components and their interactions to establish fault models, whereas ref. [29] highlighted that monitoring and control terminals act as the interfaces between cyber and physical spaces, proposing a cellular automata-based CPS risk propagation model. However, existing studies have not captured the cross-domain propagation process of cyber attacks under DG cluster control, nor have they accounted for the heterogeneity between master and slave DGs, limiting their applicability to distribution network.

Other works have explored fault impact analysis through coordination of generation, storage, and switching actions to minimize load shedding [30,31,32]. Ref. [33] decomposed the functions of smart substations into logical nodes, logical connections, and system software to evaluate risk by calculating failure probabilities and functional loss values. In addition, ref. [34] adopted Markov chains and depth-first algorithms to compute attack success probabilities, while quantifying physical consequences through weighted indices of load loss, user outage, and outage duration. However, these approaches still rely heavily on the calculation of attack success probability and simplified quantification of physical consequences. Nevertheless, whether these fault impact analysis methods remain applicable under the DG cluster control of distribution networks has not been thoroughly investigated.

In summary, existing risk assessment studies depend primarily on calculating the probability of attack success and quantifying physical consequences. Yet, under CPS-based DG cluster control, the cross-domain propagation patterns of cyber attacks differ between master and slave DGs, requiring more refined probabilistic modeling. Moreover, the physical consequences are not limited to control costs and load-shedding impacts but also include alterations in power flows and voltage within the distribution network. Therefore, current assessment methods are restrictive when applied to DG cluster control in active distribution networks. To overcome the inadequate treatment of cyber–physical threat propagation and the absence of attacker-oriented perspectives in current CPS security assessments, as well as the incomplete quantification of the security consequences induced by cyber attacks, this study constructs a CPS model for DG cluster control in active distribution networks. The model reveals the dynamic process by which cyber attacks cause cluster control failures. Based on this, a risk assessment method is proposed to evaluate DG cluster control failures under various attack paths.

The main contents of the paper include the following: Section 1, a CPS architecture and control–communication framework are established, and the cyber–physical interaction characteristics under DG cluster control are analyzed; Section 2, a logical model of cyber attack strategies targeting DG cluster inverters is developed; Section 3, a failure probability quantification method is proposed based on the control link structure and master–slave relationships; Section 4, a physical consequence evaluation mechanism is constructed by considering power deficits at cluster point of common coupling (PCC) and the results in internal network of DG cluster; and Section 5, a comprehensive CPS security assessment method is formulated and validated through simulation.

## 2. CPS Modeling and Cyber–Physical Interaction Analysis for DG Cluster Control

### 2.1. CPS Modeling of DG Cluster Control

In an active distribution network, the CPS connects the master control center and the physical grid through an information network. The CPS architecture can be divided into four layers: master station layer, substation layer, terminal layer, and physical network layer. The master station layer serves as the core of the distribution CPS, consisting primarily of the distribution master control center, as well as supporting systems such as Supervisory Control and Data Acquisition (SCADA) and Management Information System (MIS). The substation layer includes various local substations, which are connected to the master station via Synchronous Digital Hierarchy (SDH) and to terminal devices via Ethernet Passive Optical Network (EPON). The terminal layer consists of intelligent devices such as feeder terminal units (FTUs) and DG output control terminals, which are directly interfaced with electrical equipment in the physical layer.

**Figure 1:**
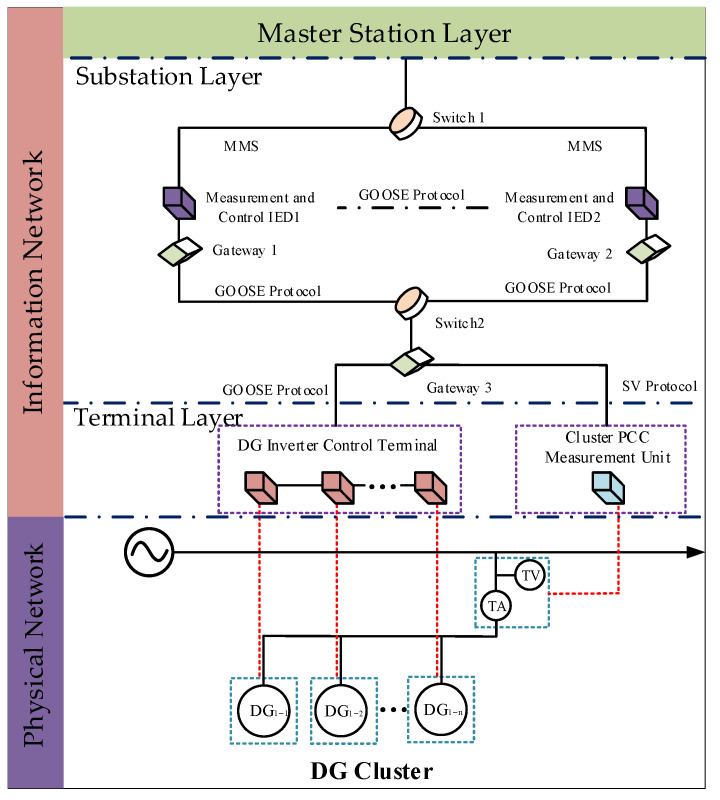
CPS model for DG cluster control in active distribution networks.

According to the IEC 61850 standard [35], a CPS model for DG cluster control can be constructed, as shown in Figure 1. The CPS utilizes an IP gateway to enable effective transmission of both upstream data and downstream commands under various communication protocols, ensuring interaction between the master control center and the DG clusters. During upstream data transmission, the output data collected at the cluster PCC is transmitted to the Intelligent Electronic Device (IED) via the Sampled Value (SV) protocol. In the downstream direction, the control commands issued by the master station are transmitted from the IED to the inverter control terminals of the DG cluster via the Generic Object Oriented Substation Event (GOOSE) protocol. Data exchange between the IED and the substation control layer is handled using the Manufacturing Message Specification (MMS) protocol. Meanwhile, interlocking data exchange between IEDs is also implemented through GOOSE messages.

### 2.2. Analysis of Cyber–Physical Interaction in DG Cluster Control

**Figure 2:**
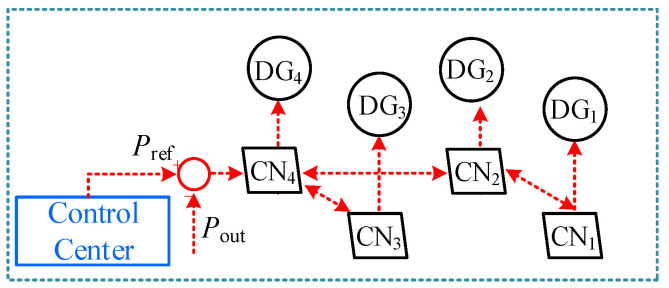
CPS architecture for DG cluster control in active distribution networks.

DG cluster control refers to the coordinated regulation of geographically adjacent DGs, as illustrated in Figure 2. The DGs are typically inverter-based resources, such as photovoltaic systems, direct-drive wind turbines, and fuel cells. Their output performance is primarily determined by the response characteristics of the inverter control system. The inverter regulates both active and reactive currents to ensure that the output power tracks the given reference value. Under DG cluster control, multiple neighboring DGs are integrated into the distribution network as a controllable unit, with their power output coordinated through the communication capabilities of the CPS.

As shown in Figure 2, the CPS of the distribution network consists of a cyber layer and a physical layer. Information nodes (CNs) within the cyber layer are interconnected via communication links, and each is also connected to its corresponding physical node through the CPS communication network. Under DG cluster control, each DG in the cluster is mapped one-to-one to a CN in the cyber layer. For example, the control center is directly connected to CN4, which corresponds to DG4. The control center sends real-time control commands to CN4, which then forwards the commands to other CNs in the same cluster—namely CN1, CN2, and CN3. These CNs subsequently transmit the commands to their respective DGs. This hierarchical communication process enables the DG cluster to track the total output power reference issued by the dispatch center. The control objective function is formulated as

(1) $$f=\underset{P}{\min}{\left(P_{\mathrm{out}}\left(P\right)-P_{\mathrm{ref}}\right)}^{2}$$

where *f* is the objective function; $P_{\mathrm{out}}$ is the total active power output from the DG cluster to the distribution network, which can be expressed as a function of the individual DG outputs P. The vector P is defined as

(2) $$P={\left[P_{DG1},P_{DG2},P_{DG3},\ldots ,P_{DGn}\right]}^{T}$$

where $P_{DG1},P_{DG2},P_{DG3},\ldots ,P_{DGn}$ represent the active power outputs of each DG unit in the cluster.

As a controllable unit, the DG cluster must not only maintain controllability of the total cluster output, but also ensure coordination among the output levels of individual DGs. Specifically, the output ratios of all DG units should remain the same across the cluster. This coordinated output constraint is given by

(3) $$\frac{P_{DG_{1}}}{P_{DG_{1},\max}}=\frac{P_{DG_{2}}}{P_{DG_{2},\max}}=\ldots =\frac{P_{DG_{n}}}{P_{DG_{n},\max}}=\xi$$

where $P_{\mathrm{DG}i,\max}$ is the maximum allowable output of $DG_{i}$; $P_{DG_{i}}$ is the actual output power of the *i*-th DG, with $0\leq P_{DG_{i}}\leq P_{DG_{i},\max}$; *n* is the total number of DG units in the cluster; ξ is the output ratio for all DG units. When the dispatch center issues a real-time control command updating the reference power $P_{\mathrm{ref}}$, $P_{DG_{i}}$ must comply with the constraint defined by (2).

The DG cluster consists of two types of units: master DGs and slave DGs. The master CN corresponds to the DGs that directly receive control commands from the control center. The slave CNs correspond to the DGs that receive control information indirectly from the master CN. In Figure 2, DG4 is the master DG, while DG1, DG2, and DG3 are slave DGs. At time t, the information node CN4 of DG4 receives the real-time control command $P_{\mathrm{ref}}\left(t\right)$ issued by the control center, as well as the actual cluster output power $P_{\mathrm{out}}\left(t\right)$ measured at PCC. Based on the current output ratio ξ(t), CN4 updates the output ratio for the next time step ξ(t+1) to meet the control objective defined in Equation (1). The ξ(t+1) is given by

(4) $$\xi (t+1)=\min\left\{\max\left[\xi (t)+\right.\right.\left.\left.\lambda (P_{\mathrm{out}}(t)-P_{\mathrm{ref}}(t))\;,0\;\right],1\right\}$$

where λ is the adjustment coefficient, and λ>0.

DG4 updates its output power at time t+1 based on the output ratio ξ(t+1) provided by its corresponding communication node CN4. The output power at time t+1 is calculated as

(5) $$P_{DG_{i}}(t+1)=\left[\xi (t+1)\right]P_{DG_{i},\max}$$

Once the master DG updates its output ratio, it sends the value ξ(t+1) to the corresponding communication nodes CN2 and CN3. These nodes then forward ξ(t+1) to their respective DG units, enabling DG2 and DG3 to compute their output power according to Equation (4). CN2 continues sending ξ(t+1) to CN1 in the same manner. After all DGs have completed their output updates, the total cluster output $P_{\mathrm{out}}(t+1)$ is updated accordingly.

In the absence of faults, this process ensures coordinated control of the total output power $P_{\mathrm{out}}$ at the cluster PCC. The CPS communication layer transmits the control instructions issued by the dispatch center to the physical devices. Each DG adjusts its output to match the control objective, while the physical layer uploads status data to the cyber layer. This allows the dispatch center to monitor the operational state of the physical system in real time.

## 3. Cyber Attack Strategy on DG Inverters

A false data injection attack (FDIA) targeting DG inverters aims to disable the entire cluster of inverters by corrupting their information. FDIA can be categorized into two types: falsifying upstream measurement data and manipulating downstream dispatch commands. Downstream command tampering involves exploiting security vulnerabilities in network components to tamper control instructions issued by the dispatch center. These tampered commands are then relayed across communication nodes within the network, resulting in erroneous dispatch actions. Upstream measurement data attacks target the communication link between the measurement unit at the cluster PCC and the physical network. By tampering with the actual data reported upstream, the attacker misleads the dispatch center into issuing incorrect control commands [31].

**Figure 3:**
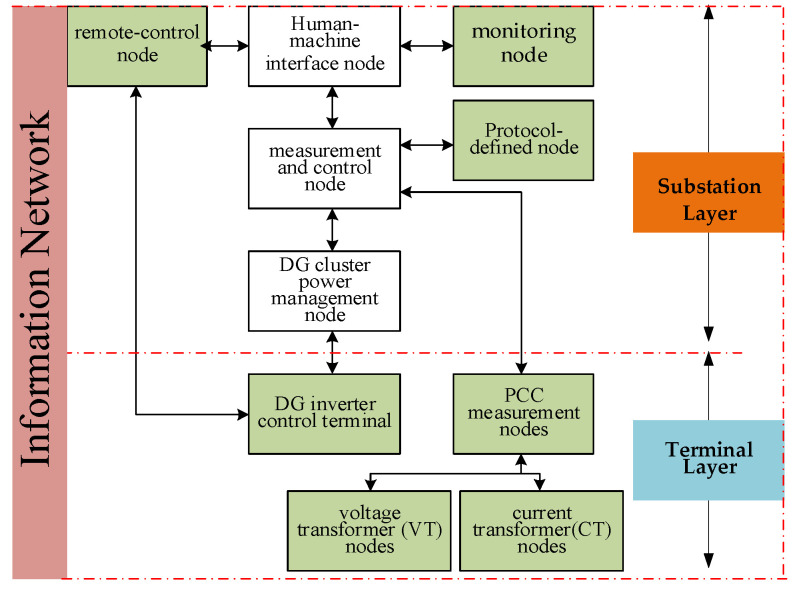
Communication logic model of CPS for DG cluster control.

**Figure 4:**
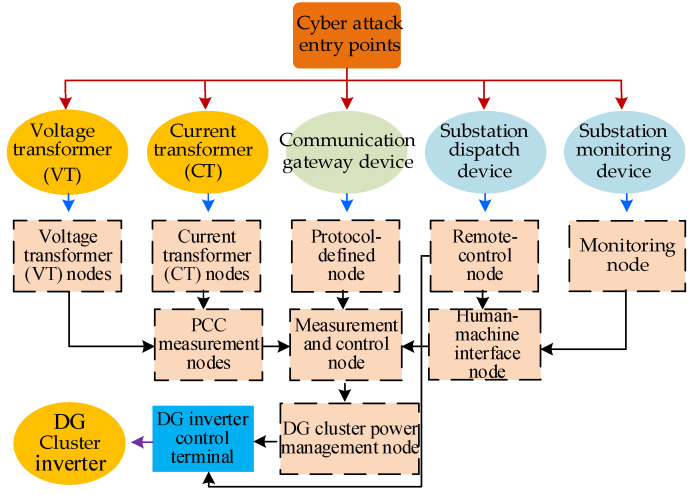
Cyber attack strategies targeting DG cluster inverters.

The SDH-based optical connection between the master station and the substation layer is generally secure and stable. Due to its high robustness, SDH communication is rarely selected as an attack target. Therefore, this study focuses on attacks occurring between the substation and terminal layers. As shown in Figure 3, the CPS model for DG cluster control includes a communication structure between the substation and terminal layers, detailed in Figure 4. Nodes in the substation layer include the human–machine interface node, monitoring node, remote-control node, measurement and control node, and DG cluster power management node. Nodes in the terminal layer include the DG inverter control terminal and the PCC measurement nodes, such as current transformer (CT) and voltage transformer (VT) nodes. Data exchange between the substation and terminal layers is realized via EPON. However, EPON suffers from weak intrinsic security, low-grade firewalls, and a lack of comprehensive intrusion detection, making it vulnerable to cyber attacks within the CPS architecture.

Under the MMS protocol, data transmission lacks encryption and user authentication mechanisms, making it highly vulnerable to cyber attacks. If the MMS protocol stack is compromised, the associated IEDs (Intelligent Electronic Devices) may crash. When a large number of IEDs fail simultaneously, it may result in the paralysis of the entire communication network. Both GOOSE and SV protocols use a publisher–subscriber model, requiring low communication latency. Moreover, their data encoding format—ASN.1—is transmitted in plaintext. As a result, the communication networks based on GOOSE and SV are vulnerable to attacks such as FDIA and GPS spoofing. These attacks tamper data content or delay signals, undermining the CPS’s ability to respond correctly to physical-layer control objectives.

Security vulnerabilities also exist within the CPS’s information and physical components. For example, monitoring and remote dispatch devices in the substation control layer often lack reliable protection against cyber intrusions. Once a substation control device is compromised, attackers can escalate privileges to access other information components, eventually disrupting the DG cluster control system. Additionally, measurement units at the cluster PCC in the physical layer are frequently exploited as entry points for attacks. Due to insufficient physical isolation, these units are vulnerable to data manipulation. Attacks on these devices can inject false measurement data into the CPS, causing the dispatch center to issue incorrect control commands.

In Figure 3, the green modules represent communication nodes with security vulnerabilities, which are more likely to be targeted by cyber attacks. Based on the CPS model for DG cluster control in active distribution networks and the corresponding communication logic model, a set of cyber attack strategies targeting DG inverters can be constructed. The logical framework of these strategies is illustrated in Figure 4. There are five possible entry points for cyber attacks on DG inverters. These include three vulnerable components within the communication network—namely, the substation monitoring device, substation dispatch device, and communication gateway device—as well as two vulnerable components within the physical network: the current transformer (CT) and voltage transformer (VT) located at the cluster PCC.

The attack entry points are indicated by the red arrows in Figure 4. If the cyber attack succeeds, the hacker gains access privileges to the compromised communication node, as marked by the blue arrows. The attacker then continues to escalate permissions by targeting additional communication nodes. Through this process, the attack state (falsified data or control commands) is propagated across the communication network. The transition of the attack state toward the DG inverter control terminal is represented by black arrows. Once the attack state reaches the target node as indicated by purple arrows, the DG inverters fail, resulting in the failure of the DG cluster control system, The entire process represents a cross-domain attack chain from the cyber domain to the physical domain. It begins with exploiting security vulnerabilities in cyber devices, propagates the attack state through the communication layer, and ultimately disrupts the operation of the target device, causing adverse impacts on the physical system.

## 4. CPS Security Assessment Method for Active Distribution Networks

### 4.1. Failure Probability Model of DG Cluster Control

As shown in Figure 4, five types of devices with security vulnerabilities serve as potential entry points for cyber attacks. These entry points represent five distinct categories of attack resources. In the false data injection attack, attackers infiltrate RTUs, SCADA/EMS, or communication nodes, using privilege escalation and data manipulation tools. However, the attacker’s computational power, encryption capabilities, and packet processing are limited. The number of communication links available for intrusion is finite, and excessive data tampering can reduce attack stealth. These constraints determine the total available attack resources, which are modeled by the computational, communication, and data injection costs required for tampering with measurement points or communication nodes. Let G denote the complete set of attack deployment strategies. Each element $g_{i}\in G$ corresponds to a specific deployment strategy of attack resources, and can be defined as

(6) $$g_{i}=\left\{c_{i,1},c_{i,2},\ldots ,c_{i,n_{s}}\right\}$$

where i denotes the *i*-th attack resource deployment strategy and $i=1,2,\ldots ,n_{w}$; $n_{w}$ is the total number of possible attack deployment strategies; $j=1,2,\ldots ,n_{s}$; $n_{s}$ is the total number of vulnerable devices that can serve as attack entry points; $c_{i,j}$ represents the amount of attack resources allocated to the *j*-th entry-point device under the *i*-th attack deployment strategy.

The probability that a specific entry-point device is successfully attacked is denoted by $p_{i,j}$. The probability that a state change in the specific device causes the failure of the target communication node is denoted by $p_{ROADj}$. The probability that the attack on the specific device leads to the failure of the target communication node is denoted by $p_{j}$. The total failure probability of the DG cluster control is denoted by p [25].

Under the *i*-th attack resource deployment strategy, the total amount of attack resources allocated to the *j*-th entry-point device is given by

(7) $$C_{i}=c_{i,1}+c_{i,2}+\ldots +c_{i,n_{s}}$$

(8) $$0\leq c_{i,j}\leq C_{i}$$

where *C_i_* is the total amount of attack resources available under the *i*-th deployment strategy.

The success of cyber attacks depends on the vulnerabilities of the entry-point device, and the exposure time of these vulnerabilities directly impacts the exploitability of the vulnerabilities. Therefore, considering the impact of time scale on the exploitability of vulnerabilities, the Pareto distribution is used to describe the effect of vulnerability exposure time on the exploitability probability of the entry-point device. The greater the exploitability of the vulnerabilities, or the more attack resources deployed at the entry-point device, the easier it is for attackers to capture the vulnerability and complete the attack. Therefore, the probability that a specific entry-point device is successfully attacked is defined as

(9) $$p_{i,j}=c_{i,j}{\left(1-\frac{k}{t_{j}}\right)}^{\alpha }$$

where k and α are the parameters of the Pareto distribution; $t_{i,j}$ denotes the exposure time of the *j*-th entry-point device; $p_{i,j}$ denotes the probability that the *j*-th entry-point device is successfully attacked under the *i*-th deployment strategy and $0\leq p_{i,j}\leq 1$.

Under the *i*-th attack resource deployment strategy, the more attack resources allocated to the *j*-th entry-point device, the higher the probability of a successful attack. Once the device is compromised via its vulnerability, the attack state is propagated through the communication network. To propagate the attack state across multiple intermediate communication nodes and eventually reach the target node, more access privileges are required.

As shown in Figure 4, the probability that the attack state successfully reaches and disables the target communication node is related to both the robustness of the communication path and the number of attack resources allocated:

(10) $$p_{ROADj}=1-e^{-\frac{c_{i,j}}{\beta_{j}}}$$

where $p_{ROADj}$ represents the probability that the target communication node becomes invalid due to the propagation of the attack state along communication path *j*; $\beta_{j}$ is the robustness factor of communication path *j*, $\beta_{j}>0$.

**Figure 5:**
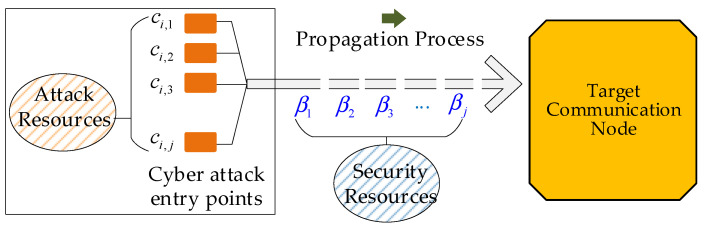
Framework of cyber attack propagation considering attack resources and robustness factors.

Equation (10) can be explained using the conceptual diagram shown in Figure 5. As illustrated, the probability that an attack state propagates successfully to the target communication node is jointly determined by the attack resource deployment strategy $c_{i,j}$, and the robustness factor $\beta_{j}$.

The determination of $\beta_{j}$ is based on the deployment decisions for the security resources of the cyber system. The decision-making scheme of the security resources can be established through AHP and KL-TOPSIS methods, which involve constructing an objective layer, first-level criteria layer, second-level criteria layer, and decision layer. The objective layer consists of robustness factor values. The first-level criteria layer includes four primary indicators: industrial control network environment, industrial control communication protocols, device node security, and encryption algorithm strength. The second-level criteria layer comprises 12 secondary indicators, such as firewall configuration, information confidentiality, and identity authentication. The decision layer involves making decisions on the type and level of defense resources against the propagation of attack states.

According to the AHP method, pairwise comparisons are made among the indicators in the first-level criteria layer to determine the judgment matrix:

(11) $$\begin{array}{cc} O= & \begin{array}{cc} & \begin{array}{cccc} O_{1} & O_{2} & \ldots & O_{n} \end{array}\\ \begin{array}{c} O_{1}\\ O_{2}\\ \vdots \;\\ O_{n} \end{array} & \left[\begin{array}{cccc} o_{11} & o_{12} & \ldots & o_{1n}\\ o_{21} & o_{22} & \ldots & o_{2n}\\ \vdots \; & \vdots \; & & \vdots \;\\ o_{n1} & o_{n2} & \ldots & o_{nn} \end{array}\right] \end{array} \end{array}$$

where $O_{n}$ represents the *n*-th indicator; $o_{mn}$ represents the importance value of indicator *m* compared to indicator *n* in the first-level criteria layer, determined using the 9-point scale method, as shown in Table 1:

Based on the judgment matrix, the weights of the indicators are calculated. The average value of each row in the judgment matrix is computed, and the arithmetic mean is defined as

(12) $${\bar{w}}_{n}=\frac{1}{m}\overset{m}{\underset{j=1}{\sum }}o_{nj}$$

Next, the average values of each row are normalized to obtain the weights, that is

(13) $$w_{n}={\bar{w}}_{n}/\overset{m}{\underset{k=1}{\sum }}{\bar{w}}_{n}$$

Similarly, the weights of the indicators in the second-level criteria layer are calculated, thereby obtaining the weight matrix from each second-level indicator to the objective layer, where each element is

(14) $$f_{n}=w_{n}v_{n}$$

where $v_{n}$ is the weight of the *n*-th indicator in the second-level criteria layer; $w_{n}$ is the weight of the first-level criteria layer indicator corresponding to the *n*-th indicator in the second-level criteria layer.

The KL-TOPSIS method is used to calculate the vulnerability factor based on the weight matrix. Let there be M defense decision variables, and N evaluation indicators in the second-level criteria layer. The value of the *n*-th indicator for the *j*-th defense decision variable is denoted by $x_{jn}$ (*j* = 1, 2, …, M; *n* = 1, 2, …, N). The decision matrix is given by

(15) $$X_{M\times N}=\left[\begin{array}{cccc} x_{11} & x_{12} & \ldots & x_{1n}\\ x_{21} & x_{22} & \ldots & x_{2n}\\ \vdots \; & \vdots \; & & \vdots \;\\ x_{j1} & x_{j2} & \ldots & x_{jn} \end{array}\right]$$

The decision matrix is normalized as follows:

(16) $$y_{jn}=\frac{x_{jn}}{\sqrt{\sum_{j=1}^{M}x_{jn}^{2}}}$$

Then, construct the weighted normalized matrix Z and calculate the positive ideal solution (PIS) and negative ideal solution (NIS)**:**

(17) $$\begin{array}{l} z_{jn}=f_{n}y_{jn}\\ \left\{\begin{array}{l} z_{n}^{+}=\underset{j}{\max}(z_{jn})\\ z_{n}^{-}=\underset{j}{\min}(z_{jn}) \end{array}\right. \end{array}$$

where $z_{jn}$ represents the elements of Z; $f_{n}$ represents the *n*-th element of the weight matrix for the second-level indicators to the objective layer; and $z_{n}^{+}$ and $z_{n}^{-}$ represent positive ideal solution and negative ideal solution, respectively.

Based on the KL-TOPSIS evaluation system, the KL distance between each decision and the positive ideal solution is calculated as

(18) $$\begin{array}{l} d_{j}^{+}=\overset{N}{\underset{n=1}{\sum }}\left[z_{n}^{+}\mathrm{lg}\frac{z_{n}^{+}}{z_{jn}}+(1-z_{n}^{+})\mathrm{lg}\frac{1-z_{n}^{+}}{1-z_{jn}}\right]\\ d_{j}^{-}=\overset{N}{\underset{n=1}{\sum }}\left[z_{n}^{-}\mathrm{lg}\frac{z_{n}^{-}}{z_{jn}}+(1-z_{n}^{-})\mathrm{lg}\frac{1-z_{n}^{-}}{1-z_{jn}}\right] \end{array}$$

Calculating the closeness coefficient of each decision object to the ideal solution, the vulnerability factor is computed by averaging the expert scores for each step:

(19) $$\beta_{j}=\frac{(d_{j}^{+}+d_{j}^{-}){\bar{x}}_{j}}{d_{j}^{-}}$$

where ${\bar{x}}_{j}$ is the average score of all evaluation indicators for the *m*-th decision object.

According to Equation (10), a smaller robustness factor $\beta_{j}$ for communication path *j* leads to a higher probability that the target communication node becomes invalid due to the propagation of the attack state. Similarly, allocating more attack resources to communication path *j* also increases this probability. Based on the deployment of attack resources to vulnerable devices, the probability that the target communication node becomes invalid—and thereby causes the failure of the DG cluster control—is given by

(20) $$p_{j}=p_{i,j}p_{ROADj}$$

where $p_{j}$ is the probability that, under the *i*-th attack resource deployment strategy, a successful attack on the *j*-th entry-point device causes failure of the target communication node.

By summing the failure probabilities across all communication paths, the total failure probability of the DG cluster control under the *i*-th deployment strategy is given by

(21) $$p=\sum_{j=1}^{n_{s}}p_{j}$$

Due to the limitation of total attack resources and the exclusivity of resource allocation among devices, p always satisfies 0<p<1.

### 4.2. Physical Consequence Assessment Model of DG Cluster Control Failure

The master DG serves as a regional hub, resulting in a biased allocation of defense resources in the CPS-based DG cluster control of the distribution network. Most of the defense resources are deployed for the master DG, while the slave DGs receive relatively fewer protections. Accordingly, cyber attacks that aim to disrupt DG cluster control can be classified as either targeting the master DG or targeting the slave DGs. Considering the control objective defined in Equation (1), the DG cluster should track the reference power output $P_{\mathrm{out}}$ as closely as possible. However, cyber attacks such as falsifying upstream measurement data or manipulating downstream dispatch commands can cause significant deviations between the actual output $P_{\mathrm{out}}$ and the reference value $P_{\mathrm{ref}}$. Therefore, the power deficit at the PCC of the DG cluster is used as the evaluation index for the physical consequences of cyber attacks.

When the inverter of the master DG is compromised by a cyber attack, the DG cluster control fails. Let $t_{0}$ be the time when the failure of DG cluster control occurs. After failure, each DG in the cluster maintains its output based on the last power ratio before failure, denoted as $\xi (t_{0}-1)$. Therefore, for $t\geq t_{0}$, the total output power of the cluster remains constant:

(22) $$P_{\mathrm{out}}(t)=P_{\mathrm{out}}(t_{0}-1)={P^{\prime }}_{out}$$

After time $t_{0}$, the output power of the cluster no longer changes and stays constant. If the dispatch command $P_{\mathrm{ref}}$ issued by the control center changes after failure, the power deficit at the PCC of the DG cluster is given by

(23) $$P_{\Delta }(t)=P_{ref}(t)-P_{out}(t)$$

When more defense resources are deployed around the master DG, cyber attackers are more likely to target slave DGs. The physical consequence is that the attacked slave DGs, along with their neighboring DGs that receive dispatch commands through them, lose control. Let $t_{0}$ denote the time when the failure of DG cluster control is caused by the attack on slave DGs. After this moment, all affected slave DGs and their neighboring DGs maintain the output ratio $\xi (t_{0}-1)$ as before failure. Therefore, for $t\geq t_{0}$, the output of the failed slave DGs is given by

(24) $$P_{DG,f}(t)=P_{DG,f}(t_{0}-1)$$

where the subscript “DG,f” refers to the set of slave DGs that are directly attacked or lose control due to receiving dispatch commands from attacked nodes. Let the number of DGs in this set be *m*, then

(25) $$P_{DG,f}={\left[P_{DG,f.1},P_{DG,f.2},P_{DG,f.3},\ldots ,P_{DG,f.m}\right]}^{T}$$

The DGs within the cluster that are not compromised can correctly receive dispatch commands and update their outputs accordingly. The output power of these normally controlled DGs at time t is expressed as

(26) $$P_{DG,N}(t)=\left[\xi_{\mathrm{DG},N}(t)\right]P_{DG,Nmax}$$

where the subscript “DG,N” denotes the set of DGs that are not compromised, and with a total of n−m DGs in this set, the vector of these DGs is expressed as

(27) $$P_{DG,N}={\left[P_{DG,N.1},P_{DG,N.2},P_{DG,N.3},\ldots ,P_{DG,N.n-m}\right]}^{T}$$

where $P_{DG,N.max}$ denotes the vector of maximum allowable output powers for the DGs in this set, and $\xi_{\mathrm{DG},N}(t)$ is the power ratio at time t computed by the master DG based on Equation (3).

Therefore, the output power vector P of all DGs in the cluster is given by

(28) $$P={\left[P_{DG,N},P_{DG,f}\right]}^{T}$$

Under a cyber attack targeting slave DGs, the power deficit of the *v*-th DG within the set of compromised slave DGs is given by

(29) $$P_{v,\Delta }=\xi_{\mathrm{DG},N}(t)P_{DG,max.v}-P_{DG,f.v}$$

where $P_{DG,max.v}$ is the maximum allowable output of the *v*-th DG under attack, and $P_{DG,f.v}$ is the actual output of this DG after the attack.

Accordingly, the total power deficit at the cluster PCC caused by the m compromised DGs is the sum of individual deficits, expressed as

(30) $$P_{\Delta }=\sum_{v=1}^{m}P_{v,\Delta }$$

The macroscopic physical consequence of a successful cyber attack is a power deficit at the cluster PCC. However, different sets of DGs within the cluster may be attacked to produce the same level of power deficit at the cluster PCC. The impact of cyber attacks on different DGs varies in terms of their effect on internal load nodes. Attacks targeting critical DGs have a more severe impact on internal loads. Therefore, it is necessary to further quantify the influence of the attacked DGs’ locations on the internal network, under the same cluster-level power deficit.

A network model is used to describe the internal topological connections within the distributed cluster. The network model consists of a set of nodes and the edges representing their connections. Let the network be denoted by G, where the set of nodes is V and the set of edges is E, such that G=(V,E). The node set is defined as V=(1,2...n). The edge set is defined as $E=(e_{ij}\left|i\in V,j\in V,e_{ij}\neq 0)\right.$, indicating the existence of a connection between node *i* and node *j*. If $e_{ij}$ = 1, a direct connection exists between the nodes; if $e_{ij}$ = 0, no connection exists between them.

The *v*-th DG access point within the set of attacked slave DGs is defined as node *v*. When the *v*-th DG experiences power deficits, the shortage directly affects the neighboring nodes. The node degree indicates the number of connections between node *v* and other nodes, and it serves as a direct measure of the importance of node *v* in the network model. Since the internal cluster network is connected in a radial topology, a DG shortage near the PCC can affect all downstream loads. In this context, betweenness centrality reflects a node’s ability to serve as a bridge along the shortest paths between other nodes. Thus, it indirectly represents the importance of node *v* by quantifying how upstream DG shortages impact downstream loads.

Betweenness centrality is defined as the ratio of the number of shortest paths passing through node *v* to the total number of shortest paths in the network. Let $BC_{v}$ denote the betweenness centrality of node *v*, defined as

(31) $$\left\{\begin{array}{l} BC_{v}=\underset{j\neq k\in V}{\sum }\frac{\sum_{l\in P_{jk}}\delta_{l}^{v}}{|P_{jk}|}\\ \delta_{l}^{v}=\{\begin{array}{c} 1,v\in l\\ 0,\mathrm{others} \end{array} \end{array}\right.$$

where l denotes the shortest path between nodes *j* and *k*; $P_{jk}$ is the set of all shortest paths between *j* and *k*, and $|P_{jk}|$ is total number of such paths. The indicator $\delta_{l}^{v}$ equals 1 if node v lies on the path l, and 0 otherwise.

Based on node degree and betweenness centrality, the overall importance of node *v* is quantified using the degree-betweenness importance index:

(32) $$C_{v}=DC_{v}+\frac{BC^{*}}{DC^{*}}BC_{v}$$

where $C_{v}$ denotes the importance of the *v*-th node where the attacked DG is located within the cluster; $DC_{v}$ and $BC_{v}$ are the degree and betweenness centrality of node v, respectively; $DC^{*}$ and $BC^{*}$ represent the maximum values of degree and betweenness centrality among all nodes in the network.

The direct physical consequence of a successful cyber attack is the occurrence of power deficit of each DG within the distributed cluster. The impact of each DG’s power deficit on the internal loads of the cluster is evaluated using betweenness centrality.

(33) $$R_{v,P}=\sum_{v=1}^{m}C_{v}P_{v,\Delta }$$

What’s more, when the *v*-th DG is attacked, the power flow inside the cluster changes. Let the change in power flow of the *i*-th line in the cluster be expressed as $\Delta S_{v}(i)$, $\Delta S_{v}(i)$ is given by

(34) $$\Delta S_{v}(i)=S_{0}(i)-S_{v}(i)$$

where $S_{0}(i)$ represents the transmission power of line *i* under normal operating conditions, and $S_{v}(i)$ represents the transmission power of line *i* after the *v*-th distributed generation is attacked.

According to the power flow equation, the change in the transmission power of line will cause a voltage change at each node. After the *v*-th DG is attacked, the consequence of voltage violation within the cluster is

(35) $$B_{v,V}=\rho \overset{N_{D}}{\underset{i=1}{\sum }}\frac{\exp\left(\max\left(\frac{V_{\max}-V_{i}}{V_{N}},\frac{V_{i}-V_{\min}}{V_{N}}\right)\right)-1}{e-1}$$

where $B_{v,V}$ represents the consequence of voltage violation within the cluster; $N_{D}$ represents the total number of nodes; ρ represents the dimensionally unified parameter for node voltage risk; $V_{i}$ represents the voltage at node *i* when the *v*-th DG is attacked; and $V_{\max}$, $V_{\min}$, $V_{N}$ represent the maximum voltage, minimum voltage, and rated voltage at node *i*, respectively.

Accordingly, the physical consequence of a successful cyber attack on the internal network, considering both the active power deficit and voltage violation consequence within the cluster, can be expressed as

(36) $$B_{v}=B_{v,P}+B_{v,V}$$

Cyber attacks targeting the master DG cause the distribution center of the CPS to lose control over the entire DG cluster, leading to severe physical consequences. In contrast, attacks on slave DGs only result in partial control failure within the cluster, with relatively minor physical consequences. Considering the deployment of defense resources in the CPS, the communication link associated with the master DG typically has a higher robustness factor. Therefore, attacks on the master DG have a lower success rate, while attacks on slave DGs are more likely to succeed. As a result, the overall risk of DG cluster control failure must be evaluated by jointly considering both the success probability of the attack and its corresponding physical impact. Therefore, the risk index R of DG cluster control failure can be expressed as

(37) $$R=p\left(P_{\Delta }+B_{v}\right)=pP_{\Delta }+pB_{v}$$

where p is the total failure probability of DG cluster control under the *i*-th attack resource deployment strategy; and $P_{\Delta }$ is the total active power loss at the DG cluster PCC.

The first term in the risk index represents the product of the attack success probability and the active power loss at the cluster PCC. It quantifies the severity of physical consequences at the DG cluster output caused by distributed control failure, characterizing the external power deficit risk under cyber attacks. The second term combines the attack success probability, the impact of each DG’s power deficit on the internal loads and voltage violation risks. It reflects the internal risk under cyber attacks.

## 5. Case Study

**Figure 6:**
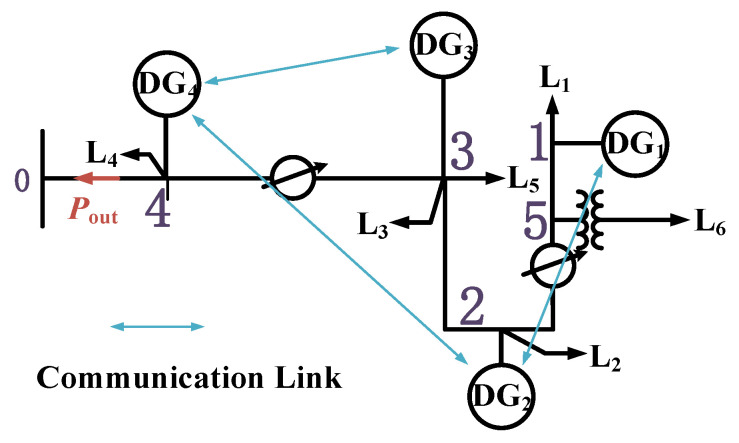
Simulation system of the distribution network CPS.

An active distribution network, as shown in Figure 6, is constructed to validate the effectiveness of the proposed CPS security assessment method. The distribution network is modeled at a voltage level of 10 kV with line impedances of 0.23 Ω/km and 0.41 Ω/km for resistance and reactance, respectively. The distribution network includes four DG units—DG1, DG2, DG3, and DG4—which form a DG cluster. The network operates under DG cluster control to regulate the power output of the cluster through node 0. DG4 serves as the master DG, while DG1, DG2, and DG3 act as slave DGs. The four DGs are connected via an information network that supports the communication structure described in Figure 1. Each DG has a maximum power output limit of 5 MW. The DG cluster supplies six loads, denoted as L_1_, L_2_, L_3_, L_4_, L_5_, L_6_, with power demands of 3 MW, 2 MW, 2.5 MW, 1.5 MW, 3.5 MW, and 2 MW, respectively. The loads are represented as constant power demands. The update interval for the DG cluster control is set to 0.1 s.

### 5.1. Failure Probability Calculation of DG Cluster Control

As shown in Figure 6, there are seven devices with security vulnerabilities that can serve as attack entry points. These include one scheduling device at the substation control layer, one monitoring device at the substation control layer, three communication gateway devices, one voltage transformer, and one current transformer. The exploitability of each vulnerability is calculated based on its exposure time, and the attack resource allocation is quantified accordingly. The detailed results are shown in Table 2. Then according to Equation (9), the probability of a successful attack on each device $p_{i,j}$ is p=[0.125,0.121,0.145,0.146,0.146,0.146,0.116].

Using the AHP and KL-TOPSIS methods [32], the robustness factors β for the seven communication links—each corresponding to one of the seven vulnerable devices—can be obtained. Substituting these values into Equation (10), the failure probability $p_{ROAD}$ of each target communication node can be calculated:

When the communication node corresponding to DG4 is selected as the target, β=[82.33,76.95,52.03,52.03,52.03,32.59,32.59], $p_{ROAD}=[0.012,0.013,0.019,0.019,0.019,0.030,0.030]$.

When the communication node corresponding to DG2 is selected as the target, β=[59.12,55.66,37.72,37.72,37.72,21.10,21.10], $p_{ROAD}=[0.017,0.018,0.026,0.026,0.026,0.046,0.046]$ When the communication nodes corresponding to DG1 and DG3 are selected as the targets, β=[41.11,36.98,19.41,19.41,19.41,6.19,6.19], $p_{ROAD}=[0.024,0.027,0.050,0.050,0.050,0.149,0.149]$.

Under equal allocation of attack resources, the failure probabilities of DG cluster control targeting DG1, DG2, DG3, and DG4 are calculated using Equations (20) and (21) as 0.067, 0.0.28, 0.067, and 0.019, respectively. DG4, as the master DG, exhibits the lowest failure probability under the same attack resource deployment strategy. DG1, DG2, and DG3 are all slave DGs; however, the communication node associated with DG2 is responsible not only for managing DG2’s output but also for forwarding scheduling instructions from DG4. In contrast, the nodes associated with DG1 and DG3 are only responsible for managing their respective DG outputs. Therefore, DG2 plays a more critical role in the CPS, and more defense resources should be allocated to DG2 than to DG1 or DG3 in actual engineering practice. As a result, under equal deployment of attack resources, the failure probability of DG2 is lower than that of DG1 and DG3.

The deployment of attack resources is subject to constraints on computation time and system load, ensuring that the allocation avoids excessive delays and prevents over-concentration on a single target or path. Considering the differences in robustness factors among communication links, the deployment of attack resources can be optimized using a cellular network resource allocation algorithm [36] to increase the probability of DG cluster control failure under a fixed total amount of attack resources. Under the optimal resource deployment strategy, the failure probabilities of DG cluster control targeting DG1, DG2, DG3, and DG4 are 0.303, 0.257, 0.303, and 0.169, respectively. Compared with the case of uniform resource allocation, the optimal deployment strategy results in higher failure probabilities for the targeted nodes.

### 5.2. Physical Consequence Calculation of DG Cluster Control Failure

At the initial moment, the control center issues a real-time dispatch instruction of 10 MW to DG4. The initial output power of each DG is 2 MW. With a power adjustment coefficient k=0.4, the output power of each DG under DG cluster control is updated according to the dispatch instruction. The failure of DG cluster control due to cyber attacks is assumed to occur at time t=1.1 s.

When DG4 experiences control failure at t=1.1 s, all DGs within the cluster enter a state of DG cluster control failure. According to Equation (24), DG1 through DG4 maintain their output based on the power ratios at t=1.0 s. The resulting power deficit at the DG cluster PCC is 3.35 MW. When DG2 experiences control failure at t=1.1 s, DG1 also loses its control capability, as it can only receive dispatch instructions forwarded by the communication node corresponding to DG2. In this case, DG1 and DG2 maintain their outputs based on their power levels at t=1.0 s according to Equation (24), while DG3 and DG4 update their outputs at t=1.1 s based on Equation (28). According to Equation (23), the power deficit at the DG cluster PCC is 2.02 MW. When either DG1 or DG3 fails at t=1.1 s, only the failed DG loses control, while the other DGs remain under normal distributed control. The failed DG maintains its output from t=1.0s based on Equation (24), and the remaining DGs update their output according to Equation (26). In this case, the power deficit at the cluster PCC is 1.68 MW. The detailed results are shown in Table 3.

The risk index is formed by not only the power deficit at cluster PCC but also the risk imposed on internal loads within the cluster. To quantify the impact of each DG’s power deficit on internal load risk after a cyber attack, it is necessary to consider the structural position of each DG in the cluster’s network topology. Therefore, based on Figure 6, the overall importance of each DG access node is evaluated in Table 4.

Based on the analysis of Table 4, DG3 is connected to Node 3. Although DG3 operates as a slave DG of DG4, its power deficit directly affects the connected loads L3 and L5. In addition, since Node 3 is located upstream in the distributed cluster, the power deficit can indirectly impact downstream loads L1, L2, and L6 through power flow. As a result, the failure of DG3 would affect both its directly connected and some downstream loads, leading to the highest comprehensive node importance value of 2.01. In contrast, DG1 is connected to terminal Node 1, whose impact on internal loads is limited. Thus, its comprehensive importance value is only 0.98.

Meanwhile, when a DG experiences a power deficit that alters the power flow, voltage deviation risks arise at the nodes within the internal transmission network of the cluster.

Table 5 indicates that node voltage risks vary with the attacked DG. DG4 attack yields the highest risk 0.95, DG2 follows 0.92, while DG1 and DG3 show lower risks 0.88. Then, according to Equation (37), the risk indices of DG cluster control failure for different DGs under the optimal attack resource deployment strategy are obtained, as shown in Table 6.

Based on Figure 6, the calculated risk values for DG1 to DG4 are 1.27, 1.66, 1.80, and 1.49, respectively. By evaluating the security of the active distribution network CPS using the proposed risk index, it is evident that the highest potential risk arises when DG3 is targeted by a cyber attack, resulting in a maximum risk value of 1.80.

To further verify the effectiveness of the proposed method, a comparative study is conducted with the approach described in [37]. In [37], a method was proposed to evaluate the impact of cyber attacks on DG clusters. This method establishes an attack–defense game model to determine the optimal deployment of defense resources for each DG under the condition of optimal attack resource allocation. It further develops a calculation approach for attack success probability as a function of component defense resources, and quantifies the physical consequences based on the power deficit of each DG after component failure to derive a risk index. According to the simulation model in this study, the results of defense resource allocation and the probability of cyber attack success are shown in Table 7.

In this method, the physical consequence of a cyber attack is evaluated as the total power deficit of all DGs. By combining the attack success probability with the corresponding physical consequence, the risk index is obtained, as presented in Table 8.

When comparing the method in [37] with the approach proposed in this paper, it can be observed that the former only establishes an attack success probability model for components, while neglecting the propagation process of attacks from the compromised component to the target communication node. Since the robustness factors of the attack propagation paths differ between the master DG (DG4) and the slave DGs, the attack success probability for the target communication node of DG4 should theoretically differ from that of the slave DGs. However, in this method, the attack success probability for DG4 is calculated as 0.295, nearly the same as that of the other slave DGs. Furthermore, the quantification of physical consequences in the simulation is limited to DG power deficits, overlooking the impact of DG deficits on internal cluster power flows and voltage. Therefore, within this method, DG4 is identified as the most critical attack target with the most severe risk consequences, which is an unreasonable conclusion since the model neglects the differences in attack propagation between the master DG and the slave DGs.

## 6. Conclusions

This paper proposes a security assessment method for DG cluster control in active distribution network CPSs, targeting control failures caused by cyber attacks. A DG cluster control model and a corresponding communication logic model are established within the CPS architecture. The propagation mechanisms and attack paths of cyber attacks in master–slave control links are analyzed, and a logic model for inverter-targeted cyber attack strategies is developed to characterize how such attacks disrupt control coordination and power output. On this basis, a failure probability calculation method is proposed. By combining this with a power deficit propagation model at the physical layer and a node importance index, a comprehensive risk index is constructed. The index integrates attack entry points, link propagation paths, and physical consequences to evaluate the security of active distribution network CPSs under different types of DG cluster control failures.

The proposed method is practical in the simulated scenario but does not model attack detection mechanisms and only evaluates security against FDI attacks. Moreover, its adaptability to other DG control architectures remains limited, which constrains its broader applicability. Future work will focus on modeling attack detection mechanisms and considering various cyber attacks, such as DDoS and malicious code injection, as well as extending the framework to different DG control architectures to enhance generalization. The risk assessment results can guide dynamic defense deployment based on the relative risk levels of different attack targets, incorporating sensitivity analysis to further improve current defense deployment methods that rely on AHP and the empirical nature it brings.

## Figures and Tables

**Table 1:** 9-Point Scale and Its Meaning.

Scale Value	Meaning	Scale Value	Meaning
$o_{mn}$= 1	$O_{m}$ is equally important as $O_{n}$	$o_{mn}$= 1	$O_{n}$ is equally important as $O_{m}$
$o_{mn}$= 3	$O_{m}$ is slightly more important than $O_{n}$	$o_{mn}$= 1/3	$O_{n}$ is slightly more important than $O_{m}$
$o_{mn}$= 5	$O_{m}$ is significantly more important than $O_{n}$	$o_{mn}$= 1/5	$O_{n}$ is significantly more important than $O_{m}$
$o_{mn}$= 7	$O_{m}$ is strongly more important than $O_{n}$	$o_{mn}$= 1/7	$O_{n}$ is strongly more important than $O_{m}$
$o_{mn}$= 9	$O_{m}$ is extremely more important than $O_{n}$	$o_{mn}$= 1/9	$O_{n}$ is extremely more important than $O_{m}$

**Table 2:** Information of vulnerabilities.

Vulnerability ID	Exposure Time (Days)	Exploitability of the Vulnerabilities	Attack Resource Allocation	Successful Attack Probability on Devices
V1	1460	0.417 3	0.3	0.125
V2	60	0.401 6	0.3	0.121
V3	730	0.482 9	0.3	0.145
V4	1460	0.485 7	0.3	0.146
V5	2190	0.487 1	0.3	0.146
V6	2190	0.487 1	0.3	0.146
V7	1095	0.385 5	0.3	0.116

**Table 3:** Distribution of power deficits for each DG under different attack scenarios.

Attacked DG	DG1 Power Deficit/(MW)	DG2 Power Deficit/(MW)	DG3 Power Deficit/(MW)	DG4 Power Deficit/(MW)	Power Deficit at PCC/(MW)
DG1	1.68	0.00	0.00	0.00	1.68
DG2	1.02	1.00	0.00	0.00	2.02
DG3	0.00	0.00	1.68	0.00	1.68
DG4	0.90	0.80	0.80	0.85	3.35

**Table 4:** Importance of DG access nodes.

DG Access Node	Degree	Betweenness Centrality	Overall Importance
1	3	0.2571	0.98
2	4	0.6381	1.74
3	5	0.6762	2.01
4	4	0.3714	1.35

**Table 5:** Node voltage risks under cyber attacks.

Attacked DG	Voltage Deviation (p.u.)					Voltage Risks
	Node 1	Node 2	Node 3	Node 4	Node 5	
1	0.037	0.031	0.027	0.022	0.033	0.88
2	0.032	0.038	0.031	0.026	0.034	0.92
3	0.027	0.030	0.037	0.028	0.021	0.88
4	0.029	0.033	0.032	0.040	0.028	0.95

**Table 6:** Risk indices of DG cluster control failure.

Attacked DG	Cluster Control Failure Probability	Power Deficit at PCC/(MW)	Overall Importance	Voltage Violation	Risk Index
DG1	0.303	1.68	0.98	0.88	1.27
DG2	0.257	2.02	1.74	0.92	1.66
DG3	0.303	1.68	2.01	0.88	1.80
DG4	0.169	3.35	1.35	0.95	1.49

**Table 7:** Result of Defense Resource Allocation and the Probability of Cyber Attack Success.

Attacked DG	Defense Resource Allocation	Probability of Cyber Attack Success
DG1	2.0	0.315
DG2	2.5	0.285
DG3	2.0	0.313
DG4	2.4	0.295

**Table 8:** Risk index results.

Attacked DG	Optimal Attack Resource Allocation	Power Deficit at PCC/(MW)	Risk Index
DG1	1.9	1.68	0.530
DG2	3.9	2.02	0.576
DG3	2.1	1.68	0.526
DG4	2.1	3.35	0.988

## Data Availability

The data used to support the findings of this study are available from the corresponding author upon request.
